# Exploratory Investigation of Biaxial Flexural Strength and Fracture Characteristics of 3D-Printed Leucite-Reinforced BisGMA/TEGDMA Composites

**DOI:** 10.1055/s-0045-1813750

**Published:** 2026-01-14

**Authors:** Chibuzo Emmanuel Nlemorisa, Stanley C. Onwubu

**Affiliations:** 1 Federal University of Technology, Owerri School of Health Technology, Owerri, Imo, Nigeria; 2 Institute of Dentistry, Queen Mary University of London, London, United Kingdom; 3 Chemistry Department, Durban University of Technology, Durban, KwaZulu-Natal, South Africa

**Keywords:** biaxial flexural strength, 3D printing, SLA printing, composite resin, leucite glass-ceramics

## Abstract

**Objective:**

The mechanical properties of 3D-printed dental resin materials are critical for their clinical success. Unfilled resin materials used in 3D printing for dental applications often exhibit reduced biaxial flexural strengths (BFSs) due to differences in material composition and printing processes. This article aims to evaluate and compare the BFS and fractographic characteristics of SLA-printed unfilled resin (UR) and composite (C) materials, and to identify factors influencing their mechanical properties.

**Materials and Methods:**

Two experimental groups were fabricated: an unfilled resin group (URG) and a leucite-reinforced composite group (CG; 35 wt% filler). The filler percentage is in an attempt to explore the possibility of slightly surpassing an already 3D printed 30 wt% ceramic composite concentrate-resin. Disc-shaped specimens ( *n* = 20 per group) were printed using SLA and post-cured according to the manufacturer's recommendations. BFS was measured using the ball-on-ring test. Weibull analysis and scanning electron microscopy (SEM) were used to assess strength reliability and fracture features. X-ray diffraction (XRD) was used to confirm the crystalline phase of the leucite filler against International Centre for Diffraction Data (ICDD) standards. One-way ANOVA and Tukey's multiple comparisons were conducted after confirming data normality and homogeneity of variances at *p* < 0.05.

**Results:**

XRD analysis confirmed the presence of tetragonal potassium aluminum silicate (leucite) phase, aligning with ICDD reference codes. The mean BFS of the URG (228.83 MPa) was significantly higher than that of the CG (91.62 MPa). The URG exhibited brittle fracture with various hackle markings and minimal phase delamination, indicative of high flexibility and energy absorption due to increased TEGDMA ratio. The CG showed lower BFS values, with fractographic features such as porosities, minor filler particle agglomeration, and phase delamination due to settling filler particles. SEM images revealed a homogeneous distribution of filler particles in CG but also showed micro-cracks and voids that compromised its mechanical integrity. Weibull analysis revealed a higher Weibull modulus for URG (10.26) compared with CG (5.48), indicating more consistent mechanical performance.

**Conclusion:**

The URG showed significantly higher BFS than the CG, likely due to greater elastic deformation and energy absorption from its higher TEGDMA content. In contrast, the CG's lower BFS was linked to porosity and filler particle settling during printing. SEM analysis revealed challenges in achieving uniform filler distribution and adequate curing. Future studies should focus on optimizing filler properties, conversion rates, and incorporating nanofillers to enhance the flexural strength of 3D-printed dental composites.

## Clinical Implications

While 3D-printed composite materials may exhibit lower biaxial flexural strength (BFS) than unfilled resins, they still fall within acceptable limits for dental applications. This suggests that clinicians can consider SLA-printed leucite-reinforced composites for a wide range of dental applications (such as anterior or provisional restorations) not subjected to high masticatory forces especially where traditional composites or ceramics may not be ideal.

## Introduction

The development of dental materials has significantly evolved with the advent of advanced polymer composites and 3D printing technologies. 1 2 This fabrication evolution, apart from being able to produce complex geometries, is both time- and cost-effective, enabling rapid execution of multiple tasks at a time. 3 Dental composites, primarily composed of bisphenol A glycidyl methacrylate (BisGMA) and triethylene glycol dimethacrylate (TEGDMA), are widely used due to their excellent aesthetic and mechanical properties. However, optimizing these materials to enhance their mechanical strength and durability remains a critical focus of research.

Among their mechanical properties, BFS is particularly important because it reflects a material's ability to resist fracture under bending stresses, avoiding premature failure from the edges, thereby increasing the detectability of defects. Stereolithography (SLA) printing, a form of additive manufacturing, has been increasingly applied in the fabrication of dental composites. SLA utilizes a photopolymerization process where a UV laser selectively cures layers of resin to form precise, intricate structures. This method offers high resolution and smooth surface finishes, making it ideal for dental applications. 4 However, the mechanical properties of SLA-printed dental composites can be influenced by various factors, including the type of resin, filler content, and printing parameters.

BisGMA and TEGDMA are commonly used as base and diluent monomers, respectively, due to their favorable properties. BisGMA provides high viscosity, rigidity, and reduced volumetric shrinkage, while TEGDMA improves manipulation by reducing the system's viscosity and promoting the degree of conversion (DC). 5 The right combination of monomers, such as these, creates resin materials suitable for dental appliance manufacturing; however, these often lack the mechanical strength required for long-term use in high-stress areas of the mouth. To address this limitation, inorganic fillers such as glass-ceramic particles, ZrO _2_ , and SiO _2_ are incorporated into the resin matrix. 6 Leucite, a potassium aluminum silicate, is a preferred filler due to its good flexural strength and wear resistance, high hardness and thermal expansion properties, and optical properties. 7

Previous studies have explored the effects of different filler contents and particle sizes on the mechanical properties of dental composites with hybrid and nano fillers, nanofibers, fibers, nanotubes, and whiskers, conferring superior mechanical properties. 8 Kundie et al 9 reported that BisGMA/TEGDMA dental composites reinforced with glass filler particles exhibited improved mechanical properties such as impact strength. Similarly, related studies 10 11 have in their studies demonstrated the potential of improving 3D-printed resins with zirconia and other ceramic fillers. However, issues such as filler agglomeration, porosities, and incomplete polymerization can compromise the overall performance of the composite. Although there are limited studies on the reinforcement of resins using leucite, the authors have yet to investigate its potential as filler in 3D-printed (especially BisGMA/TEGDMA) composites via the stereolithography technique. In this study, we investigate the BFS and fracture characteristics of 3D-printed BisGMA/TEGDMA composite resins filled with leucite particles. We compare the mechanical properties and fractographic features of the unfilled resin group (URG) and the composite group (CG), utilizing advanced characterization techniques such as X-ray diffraction (XRD), secondary electron imaging (SEI), and Weibull analysis. By understanding the influence of filler content and 3D-printed parameters on the mechanical behavior of these composites, we aim to identify strategies for optimizing their performance for dental applications.

## Materials and Methods

BisGMA, TEGDMA, and diphenyl(2,4,6-trimethylbenzoyl)phosphine oxide (Lucirin TPO), obtained from Sigma-Aldrich Co, Dorset, UK, along with fine-size silane-coated leucite glass-ceramic particles from James Kent Ltd, England, were utilized as received. BisGMA (50 wt%) was heated to 60°C in a glass beaker to reduce its viscosity for easy decanting. TEGDMA (50 wt%) was mixed manually with Lucirin TPO (2 wt%) in a separate beaker for 20 seconds before being added to the BisGMA. The resulting mixture underwent mechanical stirring at 300 rpm for 30 minutes using a magnetic stirrer with a hot plate (MSC BASIC S46 Sigma-Aldrich, United States) at 60°C. This mixture was divided into two equal parts: one served as the unfilled resin group (URG), while the other part was combined with (35 wt%) of silanated fine-size leucite glass-ceramic particles, designated as the “composite group (CG).” Having added the leucite particles, the CG underwent manual spatulation for 20 seconds followed by mechanical mixing in a shaker mixer (Turbula Type 2T F, Basel, Switzerland) at 100 rpm for 60 minutes at room temperature to ensure uniform distribution and wetting of the fillers. Despite continuous mixing, leucite fillers tended to settle during printing, which was documented and discussed as a major limitation.

### Sample Printing

A 3D specimen measuring 14 × 1 mm for BFS testing was designed using the CAD software (Meshmixer, Autodesk, San Rafael, California, United States). The design was afterward sliced using Chitubox Basic v1.0.0 (CBD Technology Co, Ltd, Shenzhen, China) and was exported as an STL file. Subsequently, a 3D SLA printer (Elegoo Saturn from Shenzhen, China) was utilized to fabricate 40 disc specimens (20 for each group). The printing parameters included a 50-µm print layer thickness, 10 seconds exposure time, and a 0° construction angle, following methodologies outlined by Hwangbo et al (2021). 12

### Postprocessing

The printed discs underwent a cleaning process whereby they were washed in 95% isopropyl alcohol (IPA) for 5 minutes to remove residual liquid resin. Subsequently, they were rinsed under running room temperature water, air-dried for 5 minutes, and then wrapped in tin foil. The wrapped discs were left to stand at room temperature for 24 hours for additional light-free post-curing before undergoing post-curing in a 405-nm UV light polymerization chamber (FormCure, Formlabs, United States) at 60°C for 30 minutes, as per the methodology outlined by Hwangbo et al. 12 During the post-curing process, the disc specimens were flipped over after the initial 30 minutes to ensure uniform exposure to light and complete curing. The specimens were subsequently tested.

### X-Ray Diffraction Analysis

The leucite glass-ceramic particles underwent characterization using an X-ray powder diffractometer, specifically the PANalytical CubiX3 from Malvern Panalytical, the Netherlands. The characterization involved θ–2θ scan measurements conducted in standard reflection mode, employing CuKα radiation with specific wavelengths (Kα1 = 1.5405980 Å, Kα2 = 1.5444260 Å, Kβ = 1.3922499 Å). A takeoff angle of 6 degrees, a step size of θ (0.05 degrees), and a count time of 199.66 seconds were utilized during the measurements. The bulk powder scan covered a 2θ range from 5 to 70 degrees, operating at 45 kV and 40 mA. Crystal phases were identified through reference code ICDD:00–038–1423, corresponding to tetragonal leucite (Potassium Aluminum Silicate, KAlSi _2_ O _6_ ).

### Biaxial Flexural Strength Testing

Forty disc specimens (20 each for URG and CG) measuring 14 × 1 mm underwent testing to determine their BFS using the ball-on-ring test method. This involved placing each disc at the center of a 10-mm diameter support ring and covering it with a 0.05-mm plastic sheet to ensure uniform load distribution. Subsequently, a 10-KN load cell was centrally applied to each disc using a 4-mm diameter ball indenter in a universal testing machine (Instron 5567, USA, ISO 7500) at a crosshead speed of 1 mm/min. The BFS for each specimen was calculated using the Timoshenko and Woinowsky-Kreiger 13 equation:



where; *σ* = maximum tensile stress (MPa), *P* = fracture load (N), *a* = radius of the ball (mm), *h* = mean specimen thickness (mm), *v* = Poisson's ratio [ *v*_resin_ = 0.36, 14 *v*_composite_ = 0.35. 15

## Data Analysis

The strength data underwent statistical analysis employing one-way ANOVA and Tukey's multiple comparisons test at a significance level of *p* < 0.05 using Sigma Stat version 2.03 software by SPSS Inc. (Chicago, Illinois, United States). The BFS values were arranged in ascending order, and Weibull analysis was conducted on the two test groups utilizing Weibull Smith software from Fulton Findings (United States). The analysis involved plotting the double log of  against the log of the actual data using median rank regression methods, and calculated using the Weibull two-parameter distribution function 16 :



where *P _f_* is the probability of failure, *σ* is the strength at a given *P _f_* value, *σ*_0_ is the characteristic strength, and *m* is the Weibull modulus. Data sets were compared for the overlap of their double-sided confidence intervals at the 90% level to determine differences.

### Scanning Electron Microscopy

For the microscopic analysis of the composite matrix, a fragment from the CG was embedded in a block of epoxy resin (EpoxyFix, Batch no: 1161–0, Denmark) with the loading surface oriented upward. It underwent polishing using 240 grit and 2,500 grit SiC abrasives for ∼15 minutes under water irrigation, followed by 1 and 0.3 µm alumina micro polish (Buehler, United States, Lot no: 4010077), and rinsing with clean water. Two fragments from both URG and CG were affixed onto coded brass stubs using carbon sticks. These, along with the sample embedded in epoxy resin, underwent gold coating in a sputter coater (Agar Scientific Ltd, UK) for 45 seconds at 20 mA. Subsequently, they were imaged using a field emission scanning electron microscope (FEI Inspect F, Oxford Instruments, Oxfordshire, UK) in the secondary scanning imaging mode.

## Results

### X-Ray Diffraction

The X-ray powder diffraction analysis revealed diffraction peaks aligning with the ICDD reference codes 00–038–1423, indicating the presence of tetragonal potassium aluminum silicate (leucite, KAlSi2O6), as shown in Fig. 1 .

**Fig. 1:**
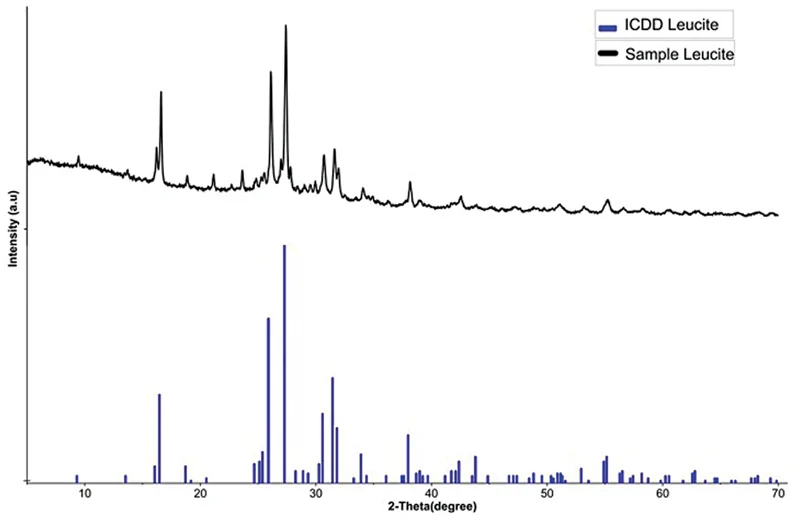
XRD patterns of the tetragonal leucite used in this study (upper) compared with the patterns of tetragonal leucite obtained from the ICDD (lower).

### Biaxial Flexural Strength

The BFS results are presented in Table 1 . The test passed (Shapiro–Wilk) normality test at 95% confidence interval with *p* < 0.001 and the critical level, *p* < 0.05. There was a statistically significant difference ( *p* < 0.001) between the mean BFS values of URG [228.83 (26.83)] and CG [91.62 (18.75)]. The results of the Weibull analysis are listed in Table 1 . The *m* value for URG (10.26) was significantly higher than that of CG (5.48). There was also a significant difference in the characteristic strengths of both sample groups according to the values of the correlation coefficient ( *r* ^2^ ) at 90% CI, which did not overlap.

**Table 1:** Biaxial flexural strengths and Weibull results

Sample	Mean biaxial flexural strength (SD), MPa	Weibull modulus, *m*	Characteristic strength, *σ*_0_ (MPa)	Regression coefficient, *r* ^2^	Confidence interval for *m* (90%)	Confidence interval for *σ*_0_ (90%)
URG	228.83 (26.83) ^a^	10.26 ^a^	239.73 ^a^	0.95	7.92–13.29	230.8–249
CG	91.62 (18.75) ^b^	5.48 ^b^	99.02 ^b^	0.90	4.1–7.34	92.26–106.3

Abbreviations: CG, composite group; URG, unfilled resin group.

Note: Superscript letters indicate significant differences ( *p* < 0.001).

The fit to the straight line was calculated using the 90% critical correlation coefficient (CCC), using Monte Carlo to approximate the distribution of the *r* ^2^ from an ideal Weibull with median rank plotting positions. It is a good fit since the *r* ^2^ was larger than the CCC and vice versa, as shown in Fig. 2A, B.

### Secondary Electron Imaging

The fracture photomicrographs of URG are shown in Fig. 3A–D, and those of CG are shown in Fig. 4(A–F). The URG displayed brittle fracture with twist hackles just below the critical flaw origin with mists and velocity hackles surrounding a mirror region, as well as presenting visibly clear corner hackles and Wallner lines in Fig. 3A . Fig. 3B revealed step hackles travelling from the tensile to the compressive surface. Fig. 3C showed the critical flaw origin in the tensile region with a crack propagating toward the compressive surface. Parallel ridges were revealed in phase delamination in Fig. 3C, D.

**Fig. 2:**
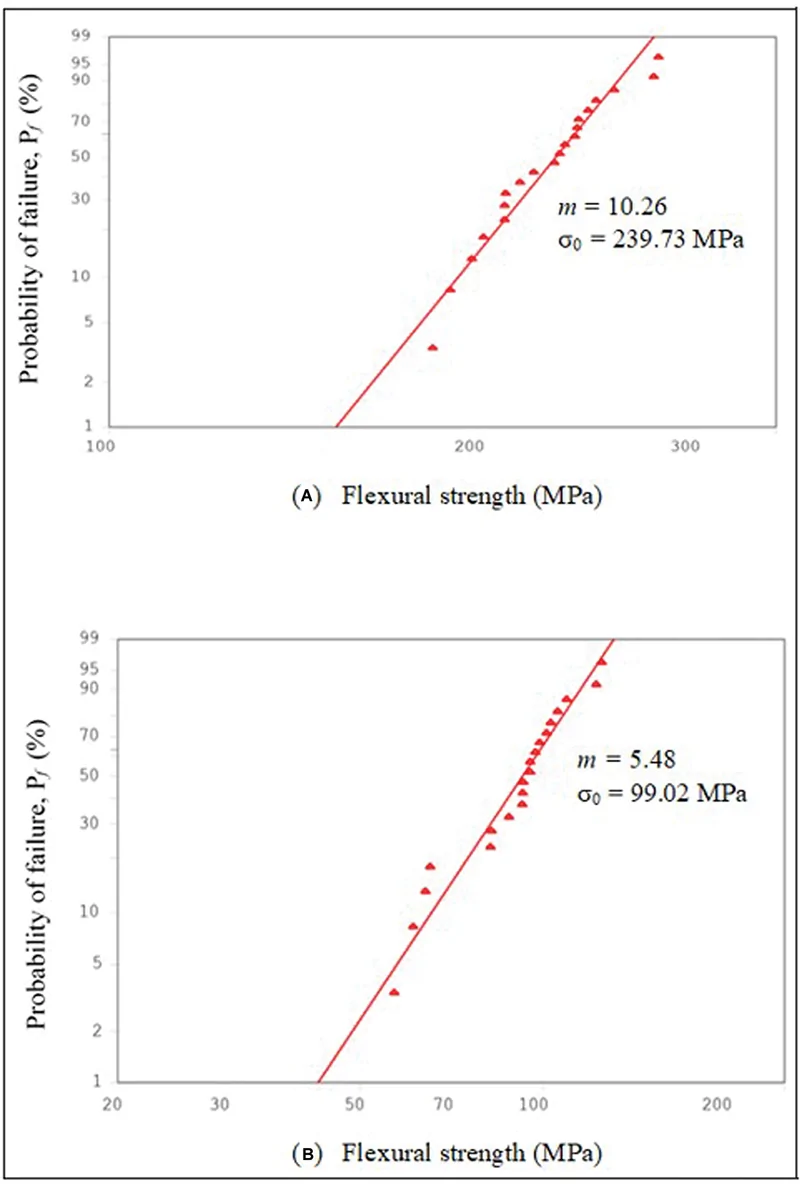
Weibull plots for biaxial flexural strength distributions for the ( **A** ) unfilled resin group and ( **B** ) composite group.

**Fig. 3:**
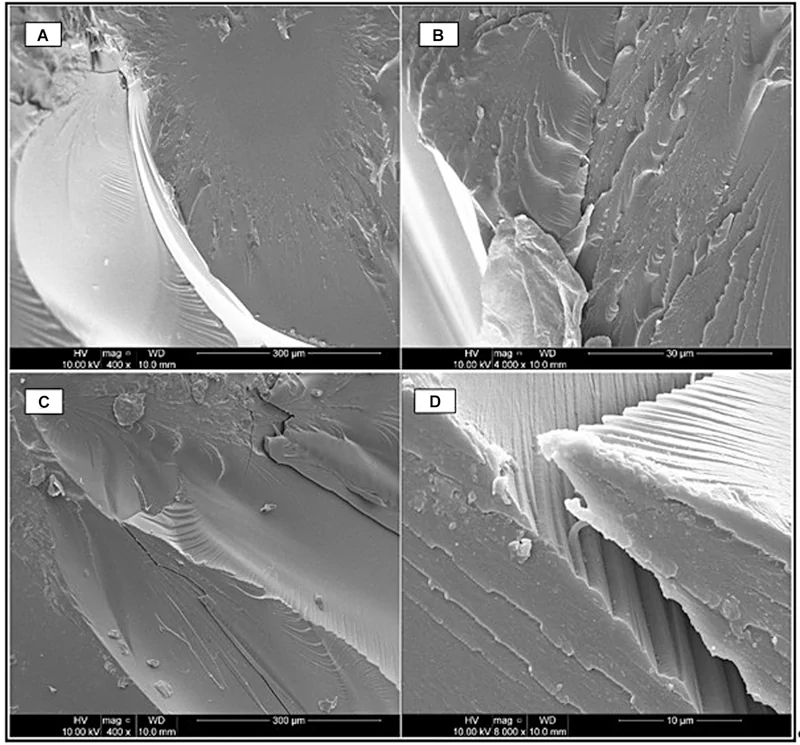
SEM photomicrographs of fractured surfaces of the unfilled resin group showing ( **A** ) mirror surrounded by mist, velocity hackles, and Wallner lines; ( **B** ) step hackles travelling from the tensile to compressive surface; ( **C** ) critical flaw origin; ( **D** ) delaminated print layers with elastic layer strand.

Pores and voids suspected to have resulted from porosities are visibly present in the composite samples with multiple wake hackles propagating toward the tensile surface in Fig. 4A , and intergranular fracture resulting in interfacial debonding in Fig. 4B . Fig. 4C shows stepping exposing filler particles, and Fig. 4D shows step fractures with phase delamination at various locations. Fig. 4E shows an overall homogenous distribution of the filler particles within the CG matrices, albeit with filler agglomerations. Micro cracks ∼2.5 µm in length and 0.05 µm in width, likely resulting from polymerisation shrinkage, are observed in Fig. 4F .

**Fig. 4:**
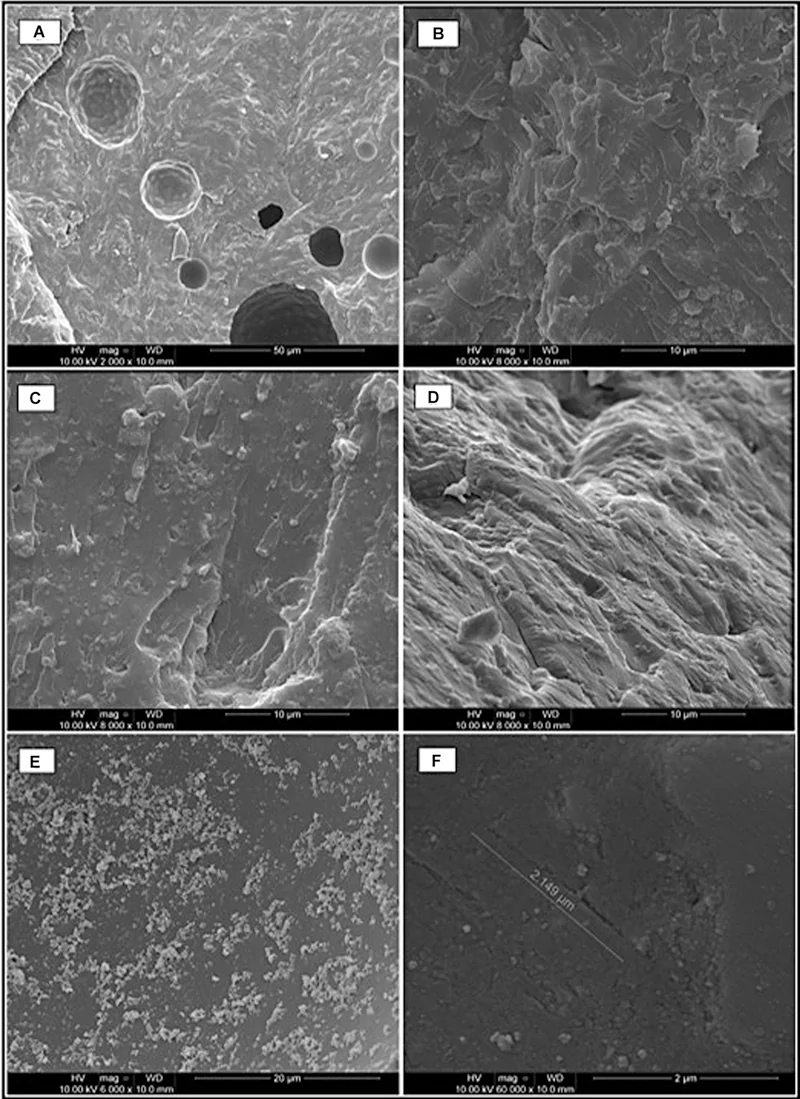
SEM photomicrographs of the composite group showing ( **A** ) voids, and wake hackles toward the tensile surface; ( **B** ) intergranular fracture with interfacial debonding; ( **C** ) stepping and exposed leucite particles; ( **D** ) step fractures and phase delamination; ( **E** ) overall filler distribution; ( **F** ) microcrack and filler agglomeration within the resin matrix.

## Discussion

The BFS results demonstrated a significantly higher strength in the URG (228.8 MPa) compared with the CG (91.62 MPa). The URG value exceeded the previously reported BFS value for BisGMA/TEGDMA neat resin (63.6 MPa) 17 and a mean BFS of 87.2 MPa for BisGMA resin, 18 but an inferior CG BFS compared with a related ZnO-reinforced resin (131.9 MPa). 17 The fractographic analysis revealed clear landmarks in the URG, while some features were obscured in the CG due to its ceramic microstructure. 19

The translucency of the URG facilitated full light transmittance during printing and post-processing, enhancing polymerization and increasing BFS. 20 The higher 50 wt% TEGDMA ratio in the URG contributed to improved flexibility, stress redistribution, and energy absorption before failure, also leading to increased BFS and reduced flexural modulus. 21 22 In contrast, the CG exhibited a more rigid but ductile nature due to filler particles trapped between the methacrylate chains. Lower BFS in composite resins has been previously linked to insufficient polymerization, weak filler–matrix interfacial bonding, and suboptimal filler characteristics. 18 23 On optimization of mechanical properties, previous studies suggest that ceramic-based composites may attain improved mechanical properties over time due to post-manufacturing polymerization. 10 This is an important phenomenon that would have augmented the CG's mechanical properties on aging. Similarly, a study by Alshamrani et al 11 demonstrated a flexural strength of zirconia glass composite ranging from 80.02 to 113.08 MPa, corroborating the findings of this study. From the highlighted evidence, it can be deduced that most ceramic fillers confer superior mechanical properties to 3D-printed resins, but at concentrations lower than 35 wt%. 10 11

One of the primary factors contributing to the reduced BFS of the CG was porosity, suspected to result from high resin viscosity, leading to crack initiation and structural weakening ( Fig. 4F ). Porosities ranging between 4 and 35 µm in diameter ( Fig. 4A ), coupled with the filler particle size being incompatible with the SLA printer resolution, further compromised mechanical integrity. 24 Additionally, the CG exhibited a heterogeneous filler distribution due to leucite particle settling, impairing light penetration and reducing the DC. 25 26 This increased polymerization shrinkage, a key factor in crack initiation 27 leading to irregular BFS values and a loose fit in the Weibull plot ( Fig. 2B ), indicating the CG's lesser reliability than the URG. 21

Despite weaker interlayer bonding in URG, evidenced by phase delamination ( Fig. 3D ), the adhesive interactions between leucite fillers and the resin matrix mitigated phase delamination and facilitated stress transfer, leading to stepping ( Fig. 4D ) and a more controlled failure mode in the CG. 23 28 According to Lee et al, 29 the loosely integrated layers of the URG, apart from encouraging delamination, could confer anisotropic behavior which ultimately increased the overall BFS, explaining partly the extreme BFS of the URG as opposed to the CG.

Although leucite particles were evenly distributed across the CG matrix ( Fig. 4E ), the effective filler percentage was likely lower than the intended 35 wt% due to leucite particle settling. Given that low-fill composites (<50 vol%) have reduced flexural strength, 30 it can be inferred that this was a significant factor in CG's BFS reduction. 31 However, deflection mechanisms, particularly stepping, helped in arresting crack propagation and bifurcation ( Fig. 4C, D ), offering some structural resilience in the CG. 28 Thus, while both groups show values comparable to provisional composites, the CG is not suitable for high-load clinical applications without further optimization.

### Limitations

One limitation of this study is the limited number of tests conducted, leaving several questions unanswered. The inability to maintain homogeneous filler distribution during printing likely compromised filler dispersion and efficient light irradiation. Additionally, viscosity-related challenges affected fluidity, reducing crosslinking and, ultimately, mechanical properties of the CG. 32

### Future Research Recommendations

Future studies should explore the impact of filler characteristics, DC, and processing parameters on mechanical properties. Research on nano-scale leucite fillers is particularly promising, as nanoparticles have been shown to enhance crosslinking, translucency, and flexural strength while reducing volumetric shrinkage. 28 31 33 Additionally, nanoparticles offer a significantly greater surface area to volume ratio compared with larger particles or bulk materials, and a greater filler loading fraction, thereby reducing volumetric shrinkage and enhancing surface hardness 30 among other mechanical properties. Given the above insight, nanoscale leucite could be a promising reinforcement in 3D printing applications in dentistry. Optimizing BFS in 3D-printed composites will require determining precise exposure times for effective C = C bond conversion using Fourier transform infrared (FTIR) spectroscopy, as suggested by Fei et al. 24 This would allow assigning a suitable and custom workflow for greater crosslinking with respect to feedstock compositions.

## Conclusion

This study demonstrated that the URG exhibited significantly higher BFS compared with the CG, although both materials performed within acceptable limits for dental applications. The reduced BFS of the composite was primarily linked to filler settling, polymerization shrinkage, and porosity formation. These factors compromised interfacial bonding and mechanical integrity, contributing to crack propagation and phase delamination (including the neat resin). Additionally, although horizontal printing is known to confer mechanical advantages, SLA, owing to its layer-by-layer fabrication process, may exhibit anisotropic behavior within layers, which can lead to reduced mechanical strength. While BFS values fell within ranges reported for provisional composites, clinical adoption of leucite-filled SLA composites requires optimization of filler suspension stability, curing protocols, and overall material formulation. By addressing the limiting factors of this study, it may be possible to develop 3D-printed leucite-dental composites with superior strength, durability, and clinical reliability.

## References

[1] Aly P Mohsen C Comparison of the accuracy of three-dimensional printed casts, digital, and conventional casts: an in vitro study Eur J Dent 2020 14 02 189 193 10.1055/s-0040-1705243 PMC7274830 32311750

[2] Noorani R 3D Printing: Technology, Applications, and Selection. 1st ed. Vol. 86 CRC Press 2017 2 12

[3] Daher R Krejci I Ardu S Time-and cost-effective 3-dimensional-printing workflow to rehabilitate worn dentitions: a clinical report J Prosthet Dent 2024 131 06 996 999 10.1016/j.prosdent.2023.02.013 36959005

[4] Ligon S C Liska R Stampfl J Gurr M Mülhaupt R Polymers for 3D printing and customized additive manufacturing Chem Rev 2017 117 15 10212 10290 10.1021/acs.chemrev.7b00074 PMC5553103 28756658

[5] Tan Q Li B Wang Z Chen Q Investigation of physio-mechanical properties of cross-linked Bis-GMA/TEGDMA dental resins: a molecular dynamics study Mater Today Commun 2023 35 105926

[6] Zha C Hu J Li A Nanoindentation study on mechanical properties and curing depth of dental resin nanocomposites Polym Compos 2019 40 04 1473 1480

[7] Novembre D Gimeno D Poe B Synthesis and characterization of leucite using a diatomite precursor Sci Rep 2019 9 01 10051 10.1038/s41598-019-46569-y PMC6624203 31296952

[8] Elfakhri F Alkahtani R Li C Khaliq J Influence of Filler Characteristics on the Performance of Dental Composites: A Comprehensive Review. Vol. 48, Ceramics International. Elsevier Ltd 2022 27280 27294

[9] Kundie F Azhari C H Muchtar A Ahmad Z A Effects of filler size on the mechanical properties of polymer-filled dental composites: a review of recent developments J Physiol Sci 2018 29 01 141 165

[10] Aati S Akram Z Ngo H Fawzy A S Development of 3D printed resin reinforced with modified ZrO _2_ nanoparticles for long-term provisional dental restorations Dent Mater 2021 37 06 e360 e374 10.1016/j.dental.2021.02.010 33663884

[11] Alshamrani A Alhotan A Kelly E Ellakwa A Mechanical and biocompatibility properties of 3D-printed dental resin reinforced with glass silica and zirconia nanoparticles: in vitro study Polymers (Basel) 2023 15 11 2523 10.3390/polym15112523 PMC10255304 37299322

[12] Hwangbo N K Nam N E Choi J H Kim J E Effects of the washing time and washing solution on the biocompatibility and mechanical properties of 3d printed dental resin materials Polymers (Basel) 2021 13 24 4410 10.3390/polym13244410 PMC8705711 34960960

[13] Timoshenko S Woinowsky-Kreiger S Symmetrical Bending of Circular Plates. Theory of Plates and Shells. 2nd ed New York McGraw-Hill 1959 33 104

[14] Chung S M Yap A UJ Koh W K Tsai K T Lim C T Measurement of Poisson's ratio of dental composite restorative materials Biomaterials 2004 25 13 2455 2460 10.1016/j.biomaterials.2003.09.029 14751729

[15] Singh S Katz L Penn R Tesk J The elastic properties of glass reinforced dental composites J Non-Cryst Solids 1988 102 112 116

[16] AboElhassan R G Watts D C Alamoush R A Elraggal A Biomechanical behavior and Weibull survival of CAD-CAM endocrowns with different marginal designs: a 3D finite element analysis Dent Mater 2024 40 02 227 235 10.1016/j.dental.2023.11.012 37977990

[17] Wellhäußer B Saure L M Schütt F Scherer F Wille S Kern M The impact of an MDP-containing primer on the properties of zinc oxide networks infiltrated with BisGMA-TEGDMA and UDMA-TEGDMA polymers Materials (Basel) 2024 18 01 137 10.3390/ma18010137 PMC11721492 39795782

[18] Wille S Hölken I Haidarschin G Adelung R Kern M Biaxial flexural strength of new Bis-GMA/TEGDMA based composites with different fillers for dental applications Dent Mater 2016 32 09 1073 1078 10.1016/j.dental.2016.06.009 27370994

[19] Quinn G D NIST Recommended Practice Guide: Fractography of Ceramics and Glasses [Internet]Gaithersburg, MD;2020. Accessed November 10, 2025:https://nvlpubs.nist.gov/nistpubs/specialpublications/NIST.SP.960-16e3.pdf

[20] Husna A Ashrafi S Tomal A A Tuli N T Bin Rashid A Recent Advancements in Stereolithography (SLA) and Their Optimization of Process Parameters for Sustainable Manufacturing. Vol. 7Hybrid Advances. Elsevier B.V.;2024

[21] Rodrigues Junior S A Zanchi C H Carvalho R V Demarco F F Flexural strength and modulus of elasticity of different types of resin-based composites Braz Oral Res 2007 21 01 16 21 10.1590/s1806-83242007000100003 17384850

[22] Musanje L Darvell B W Effects of strain rate and temperature on the mechanical properties of resin composites Dent Mater 2004 20 08 750 765 10.1016/j.dental.2003.11.008 15302456

[23] Fu S Y Feng X Q Lauke B Mai Y W Effects of particle size, particle/matrix interface adhesion and particle loading on mechanical properties of particulate–polymer composites Compos B Eng. 2008 39 06 933 961

[24] Fei G Parra-Cabrera C Li Y Stereolithographic 3D printing of graded porous materials via an integrated digital exposure and selective dissolution strategyCell Rep Phys Sci2023 407

[25] Oivanen M Keulemans F Garoushi S Vallittu P K Lassila L The effect of refractive index of fillers and polymer matrix on translucency and color matching of dental resin composite Biomater Investig Dent 2021 8 01 48 53 10.1080/26415275.2021.1906879 PMC8018547 33855302

[26] Kose C Oliveira D Roulet J F Pereira P Rocha M G Algorithm to predict the final color of leucite-reinforced ceramic restorations Dent Mater 2022 38 e65 10.1111/jerd.13007 36592128

[27] Cho Y H Lee I H Cho D W Laser scanning path generation considering photopolymer solidification in micro-stereolithography Microsyst Technol 2005 11(2–3)158 167

[28] Sakaguchi R L Powers J M Craig's Restorative Dental Materials. 13th ed Elsevier 2012 260 262

[29] Lee C S Kim S G Kim H J Ahn S H Measurement of anisotropic compressive strength of rapid prototyping parts J Mater Process Technol 2007 187–188 627 630

[30] Randolph L D Palin W M Leloup G Leprince J G Filler characteristics of modern dental resin composites and their influence on physico-mechanical properties Dent Mater 2016 32 12 1586 1599 10.1016/j.dental.2016.09.034 27720423

[31] Sideridou I D Karabela M M Vouvoudi ECh Physical properties of current dental nanohybrid and nanofill light-cured resin composites Dent Mater 2011 27 06 598 607 10.1016/j.dental.2011.02.015 21477852

[32] Quan H Zhang T Xu H Luo S Nie J Zhu X Photo-Curing 3D Printing Technique and Its Challenges. Vol. 5,Bioactive Materials. KeAi Communications Co.;2020 110 115 10.1016/j.bioactmat.2019.12.003 PMC6992881 32021945

[33] Curtis A R Palin W M Fleming G JP Shortall A CC Marquis P M The mechanical properties of nanofilled resin-based composites: the impact of dry and wet cyclic pre-loading on bi-axial flexure strength Dent Mater 2009 25 02 188 197 10.1016/j.dental.2008.06.003 18656255

